# Single-Cell Analysis Reveals Early Manifestation of Cancerous Phenotype in Pre-Malignant Esophageal Cells

**DOI:** 10.1371/journal.pone.0075365

**Published:** 2013-10-08

**Authors:** Jiangxin Wang, Xu Shi, Roger H. Johnson, Laimonas Kelbauskas, Weiwen Zhang, Deirdre R. Meldrum

**Affiliations:** Center for Biosignatures Discovery Automation, The Biodesign Institute, Arizona State University, Tempe, Arizona, United States of America; UMASS-Amherst/Tufts University School of Medicine, United States of America

## Abstract

Cellular heterogeneity plays a pivotal role in a variety of functional processes in vivo including carcinogenesis. However, our knowledge about cell-to-cell diversity and how differences in individual cells manifest in alterations at the population level remains very limited mainly due to the lack of appropriate tools enabling studies at the single-cell level. We present a study on changes in cellular heterogeneity in the context of pre-malignant progression in response to hypoxic stress. Utilizing pre-malignant progression of Barrett’s esophagus (BE) as a disease model system we studied molecular mechanisms underlying the progression from metaplastic to dysplastic (pre-cancerous) stage. We used newly developed methods enabling measurements of cell-to-cell differences in copy numbers of mitochondrial DNA, expression levels of a set of mitochondrial and nuclear genes involved in hypoxia response pathways, and mitochondrial membrane potential. In contrast to bulk cell studies reported earlier, our study shows significant differences between metaplastic and dysplastic BE cells in both average values and single-cell parameter distributions of mtDNA copy numbers, mitochondrial function, and mRNA expression levels of studied genes. Based on single-cell data analysis, we propose that mitochondria may be one of the key factors in pre-malignant progression in BE.

## Introduction

Esophageal adenocarcinoma (EAC) is a highly lethal cancer type and is believed to develop from esophageal epithelial cells through a series of complex, step-wise transformations at the biomolecular level [1]–[6]. Once transformed, EAC cells produce significantly higher levels of antioxidant molecules making them resistant to elevated levels of reactive oxygen species (ROS) [2]. Although recent studies have shown that the transformation sequence involves the development of hyperplasia and metaplasia caused by chronic inflammation of the squamous esophageal epithelium, followed by multifocal dysplasia, carcinoma *in situ* and, finally, invasive EAC [1], [7], [8], the detailed molecular mechanism underlying this transformation remains to be clarified.

Hypoxia plays a pivotal role in cancer [9]–[16]. As in almost all solid tumors, oxygen supply to cancer cells is greatly compromised due to the uncontrolled cell growth and inadequate development of the microvasculature. The mitochondrion, the powerhouse of the cell and the major source of adenosine triphosphate (ATP) in normal cells, is the place where oxidative phosphorylation (OXPHOS) takes place. Mitochondria have also been found to play a major role in programmed cell death, or apoptosis, and their dysfunction is associated with a variety of diseases. For example, variations in the mitochondrial DNA (mtDNA) copy number have been associated not only with different cellular physiological conditions but also with diverse changes of internal and external microenvironments [17], [18]. It has been demonstrated that mitochondria can generate increased levels of ROS during hypoxia [19], which led to the postulate that mitochondria, the primary target for oxidative damage, can function as an endogenous oxygen sensor.

One of the most important factors determining drug response and aggressiveness of tumors is the large intratumoral heterogeneity. Recent studies have shown that even cells in a clonal population or seemingly homogenous tissue exhibit substantial variability of different characteristics ranging from gene expression levels to phenotypic features [20]–[22]. It is now broadly accepted that mitochondrial heterogeneity, including variations in mtDNA copy number, DNA mutation/depletion, expression and regulation of genes encoded by mtDNA, and activity levels, is an important contributor to mitochondrial complexity and contributes to the overall cell-cell heterogeneity [23]–[25].

Most current bioanalytical techniques collect data using thousands to millions of cells, inherently providing results averaged over a large cell population. Such bulk-cell approaches could potentially miss important and valuable information when dealing with highly heterogeneous systems [26] such as cancer [27]. Therefore, the development and application of techniques capable of performing analyses at the single-cell level are critical, not only for a better understanding of core cellular processes, but also for new, more effective strategies for disease prevention, management, and treatment [28]–[31].

In this study we use two immortalized human Barrett’s esophageal epithelial cell lines CP-A and CP-C that were originally derived from patients with Barrett’s esophagus (BE) without dysplasia and with dysplasia, respectively [32]. Although both are nonmalignant epithelial cells, it was found that CP-C cells were more resistant to oxidative stress induced by bile acid (chenodeoxycholic acid (CDCA)) than CP-A, suggesting that, at least with regard to acid response, CP-C cells behave more like esophageal cancer cell lines as compared to CP-A cells [2]. In this study, we aim to elucidate potential mechanisms leading to malignant transformation in BE by quantifying differences in the way cells respond to the oxidative stress caused by hypoxia. We have applied a qPCR-based technique developed in our lab to determine the mtDNA copy number and the expression levels of mitochondrial and nuclear genes in individual cells. Utilizing single-cell analysis we distinguished differences in mtDNA copy number, mitochondrial membrane potential, and hypoxia response gene expression levels between CP-A and CP-C cells which cannot be predicted by bulk cell analysis. The application of these new methods, along with single-cell O_2_ consumption measurements [33]–[35], allowed the characterization of subtle hypoxia response differences between CP-A and CP-C cells. A better understanding of the molecular basis of EAC initiation and development will facilitate efforts to define potential therapeutic targets.

## Materials and Methods

### Cell Culture and Hypoxia Treatment

The Barrett’s esophageal epithelial cell lines CP-A and CP-C were obtained from ATCC and grown in Gibco® Keratinocyte Serum-Free Medium (SFM) cell growth medium (Invitrogen, Carlsbad, CA), supplemented with hEGF (Peprotech, Rocky Hill, NJ) at 5.0 µg/L, BPE (bovine pituitary extract) at 50 mg/L and penicillin/streptomycin solution (Invitrogen, Carlsbad, CA) at 100/100 µg/mL in a tissue-culture incubator at 37°C in humidified air with 5% CO_2_. Prior to experiments, cells were cultured in a 75 cm^2^ flask to approximately 80% confluence. Cells in G1 phase sorted with FACSAria (BD Biosciences, San Jose, CA) were used in qPCR experiments in this study. For hypoxia, CP-A and CP-C cells at 80% confluence were incubated in the keratinocyte SFM medium containing 2% (v/v) Oxyrase (Oxyrase, Inc., Mansfield, OH) at 37°C for 30 minutes, which is the optimal Oxyrase treatment time as determined previously [31]. The cells were subsequently trypsinized in 0.05% (v/v) trypsin solution containing 2% (v/v) Oxyrase at 37°C for 9 min. The trypsinization was blocked by adding Dulbecco’s Modified Eagle Medium (DMEM) (Invitrogen) supplmented with 5% fetal bovine serum (FBS) (Invitrogen) containing 2% (v/v) Oxyrase.

### Single-cell Harvesting

Single-cell harvesting (aspiration and dispensing) was performed using a micromanipulator developed by our group [36], [37] (Methods S1).

### Primer Design and Selection of Gene Target

Fragments within the hypervariable region I (HVI) in mtDNA were chosen for copy number analysis [38], [39]. Total DNA isolated from bulk samples (1×10^4^ cells) was used as template for mtDNA copy number measurement, and quantified using a Real-Time qPCR System (StepOne, Applied Biosystems, Foster City, CA) using optimized primers (Methods S1). For RT-qPCR expression level analysis, four mitochondrially encoded genes (16s rRNA, *COXI*, *COXIII, CYTBI* and four nuclear genes (28s rRNA, VEGF, MT3, and PTGES)(primers sequences as [31]) were chosen (Methods S1).

### Single-cell mtDNA Copy Number Determination

After harvesting, tubes each containing one cell suspended in 6 µL DNaST lysis buffer [26] were immediately frozen on dry ice, and then stored at −80°C until qPCR analysis. Each qPCR was run in a total volume of 10µL, containing 2 µL of the lysate from DNaST solution as a template. To determine the mtDNA copy number, the qPCR products of bulk samples were purified, quantified and serially diluted (copy number of 10^6^, 10^5^, 10^4^, 10^3^, 10^2^, 75, 50, 25, 10, 5, 1, 0) for a series of qPCR reactions. The results were used to plot a standard curve for each gene. The Ct value of each single cell was then transferred into absolute mtDNA copy number using the standard curves obtained.

### Mitochondrial Membrane Potential

Mitochondrial membrane potential (MMP) was quantified with confocal microscopy analysis using the potentiometric dye JC-1 (100 ng/mL) as fluorophore and published staining protocols [40], [41] (Methods S1).

### Single-cell Gene Expression Analysis

Single-cell RT-qPCR was conducted as previously described [31], [42].

### Data Analysis

Statistical analyses, including significance tests, were conducted using the OriginPro software package (v. 8, OriginLab, Northampton, MA). Statistical significance levels were calculated using the two-tailed non-parametric Mann-Whitney-Wilcoxon test.

## Results

### qPCR-based Method for mtDNA Copy Number Adetermination in Single Cells

Several mitochondrial DNA regions, such as HVI, have been used as targets for PCR-based mtDNA copy number analysis in bulk samples before [43]. One primer pair with the lowest Ct value, distinct negative control, sharp and distinct peaks of the amplification product in the melting curves, and the correct amplification product as confirmed by sequencing was selected for each region for further single-cell analysis (Fig. S1, *HV1*). Serial dilutions of PCR-amplified mtDNA fragment containing the HVI region was used to make qPCR standard curves for mtDNA copy number. With the selected primer pairs, the HVI region was demonstrated to have the best performance, with *R* ^2^ = 0.9925, for DNA copy number ranging from 10^2^ to 10^6^ (Fig. S2).

### RT-qPCR for Gene Expression Analysis in Single Cells

Recently we reported on a new technique enabling the isolation, purification, and reverse transcription of the total RNA from a single mammalian cell followed by the RT-qPCR analysis of the expression levels of nuclear-encoded genes [31]. Following the same procedure, we determined in individual cells the expression levels of several selected genes involved in hypoxia response, including mitochondrially-encoded *COXI*, *COXIII*, and *CYTBI* [43]–[45], and the *VEGF*, *MT3*, *ANGPTL4* and *PTGES* genes encoded by the nuclear DNA, with 16S rRNA and 28S rRNA were used as internal controls [31], [38], [46]–[48].

### Single-cell mtDNA Copy Number Analysis in CP-A and CP-C Cell Lines

**Figure 1:**
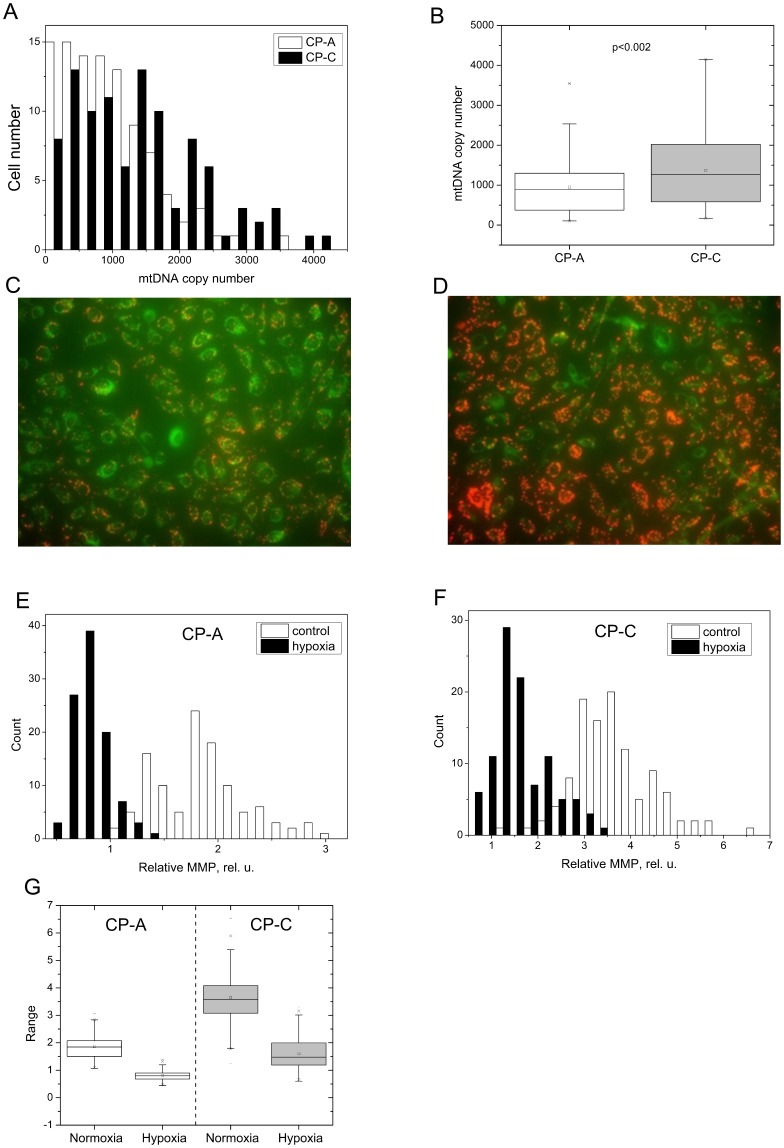
Mitochondrial DNA copy number analysis and mitochondrial transmembrane potential. A) Single cell mtDNA copy number distributions in metaplastic (CP-A) and dysplastic (CP-C) cells, B) box charts of the two distributions shown in panel A. Following values are depicted: open square – mean; solid line – median; upper and lower box lines – the 75^th^ and 25^th^ percentiles, respectively; upper and lower whiskers – the 95^th^ and 5^th^ percentiles, respectively; x – minimal and maximal values of the distribution. The p value of <0.002 was calculated using the two-tailed non-parametric Mann-Whitney statistical significance test (n = 99); mtDNA copy numbers range between 150–1,900 C–D**)** JC-1 fluorescence micrographs of hypoxic CP-A (C) and hypoxic CP-C (D) cells; single cell distributions of the relative mitochondrial membrane potential in CP-A (E) and CP-C (F) cells. JC-1 signals from about 100 cells per cell line and condition were analyzed; G) statistical representation of the MMP distribution in both cell types/conditions.

Previous studies based on bulk-cell analysis indicated that the average mitochondrial mass does not significantly differ between CP-A and CP-C cells [2]. In this study, our initial bulk-cell analysis results also showed a slightly higher but statistically not significant average mtDNA copy number in CP-C cells (∼1,530 per cell) as compared to CP-A (∼1,392 per cell) (Fig. S3). The single-cell analysis based on 99 single cells from each cell line revealed statistically significant differences (p<0.002) in mtDNA copy number between CP-A and CP-C cells (Fig. 1A, B). 92 out of 99 (92%) CP-A cells showed mtDNA copy numbers ranging between 106–1,900 per cell with only 8 (8%) cells containing more than 2,000 copies per cell, while in 92 out of 99 CP-C cells mtDNA copy numbers ranged between 171–2,952 per cell, with 8 cells having more than 3,000 copies per cell (Fig. 1A). On average, CP-C cells had about 43% more mtDNA than CP-A cells (1363 (CP-C) vs. 951 (CP-A); Fig. 1B, Table 1). Descriptive parameters of the mtDNA population histograms, including skewness (measure of symmetry), kurtosis (measure of peakedness) and the Fano factor (measure of dispersion) were calculated. Both skewness and kurtosis values were statistically significantly lower in CP-C cells as compared to CP-A cells, whereas the Fano factor was not significantly different (Table 1).

**Table 1:** Descriptive statistics of single-cell mtDNA copy number data.

Parameter	Cell type	
	*CP-A*	*CP-C*
Number of cells	99	99
Mean	951	1363
Standard deviation	678	940
SkewnessSES^a^Z value	1.190.475.09	0.790.47−0.55
KurtosisSEK^b^Z value	1.740.929.38	0.080.92−0.64
Fano factor^c^	0.71	1.14

a Standard Error of Skewness.

b Standard Error of Kurtosis.

c Fano factor is calculated as SD/Mean.

### Differential Responses to Hypoxia between CP-A and CP-C Cells at the Single-cell Level

#### Mitochondrial membrane potential differs significantly between CP-A and CP-C cells

Mitochondrial membrane potential (MMP) generated by the mitochondrial electron transport chain reflects the functional status of mitochondria. It has been linked to physiological response and the production of ROS [49]. JC-1 has been widely used in apoptosis studies to monitor mitochondrial health and dysfunction in the context of cancer and neurodegenerative diseases [40], [41].

Fluorescence imaging utilizing JC-1 staining revealed striking differences between CP-A and CP-C cells (100 cells of each type) under both control and hypoxic conditions (Fig. 1C, D). Under normoxic growth conditions (21% O_2_), CP-A cells showed relatively low red (590 nm) to green (529 nm) fluorescence intensity ratio with an average of 2, while CP-C cells exhibited an average ratio of 3.8 (Fig. 1E–G). After exposure to hypoxia for 30 min (see Methods), cells of both types showed reduced red/green fluorescence intensity ratios, averaging at 0.6 and 1.5 for CP-A and CP-C cells, respectively (Fig. 1G). The descriptive statistics data are summarized in Table 2.

**Table 2:** Descriptive statistics of MMP measurements in individual cells.

	Cell type			
Parameter	*CP-A*		*CP-C*	
	*Normoxia*	*Hypoxia*	*Normoxia*	*Hypoxia*
Number of cells	110	100	110	100
Mean	1.86	0.80	3.65	1.60
Standard deviation	0.42	0.17	0.86	0.58
SkewnessSES^a^Z value (S)	0.510.232.21	0.780.243.23	0.500.232.15	0.810.243.37
KurtosisSEK^b^Z-value (K)	0.070.460.16	1.110.482.32	0.940.462.06	0.090.480.18
Fano factor^c^	0.22	0.21	0.24	0.36

a Standard Error of Skewness.

b Standard Error of Kurtosis.

c Fano factor is calculated as SD/Mean.

Mitochondrial heterogeneity within and between different cell types was reported previously, revealing large variations in MMP among cells of the same type within a single culture dish [23]. In our study, analysis of the single-cell MMP data revealed statistically significantly higher kurtosis values in normoxic CP-C cells as compared to normoxic CP-A cells, whereas the skewness was comparable. Under hypoxic conditions we observed an opposite trend, with CP-A cells showing significantly higher values of kurtosis than CP-C cells, while skewness values were not significantly different. This data indicates differing responses to hypoxia between the two cell lines and implies that the two types of cell react to hypoxia differently, not only in terms of the average MMP, but also with regard to its distribution among individual cells. The MMP distribution kurtosis values suggest higher MMP heterogeneity of mitochondria in dysplastic (CP-C) than metaplastic (CP-A) BE cells.

#### Analysis of mitochondrial gene expression in single cells

Three mitochondrially encoded genes, *COXI*, *COXIII* and *CYTBI*, were selected for expression analysis due to their significant role in mitochondrial function and involvement in hypoxia response [44], [45]. First, we compared the relative expression levels of these genes between normoxic and hypoxic conditions in bulk cell samples using mitochondrial 16S rRNA as an internal reference. All three mitochondrial genes showed a very similar hypoxia response pattern: an increase by 1.5–2.6 fold with high variability was observed in both CP-A and CP-C cells (Fig. S4A, B).

**Figure 2:**
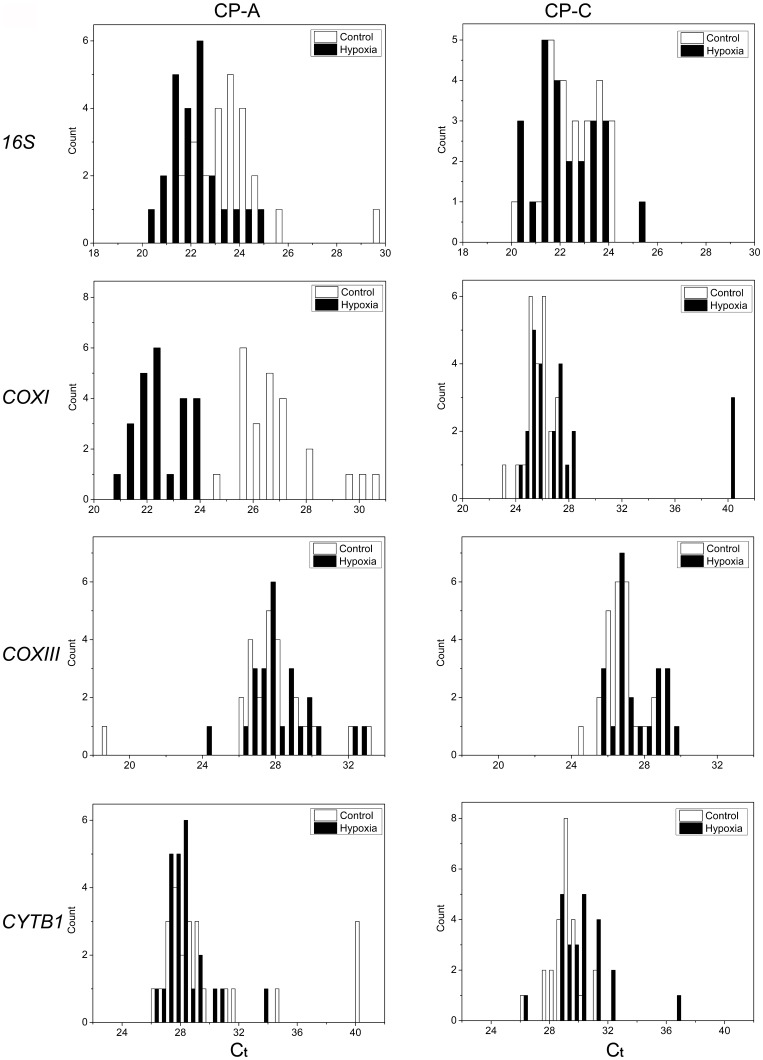
Expression levels of mitochondrially encoded genes (*16s rRNA* and *COXI*) in single control and hypoxia-treated CP-A and CP-C cells. Histograms of gene-expression levels in control (*empty bars*) and hypoxia-treated (30 minutes, *solid bars*) CP-A and CP-C cells. The distribution histograms were generated using the same bin size.

**Figure 3:**
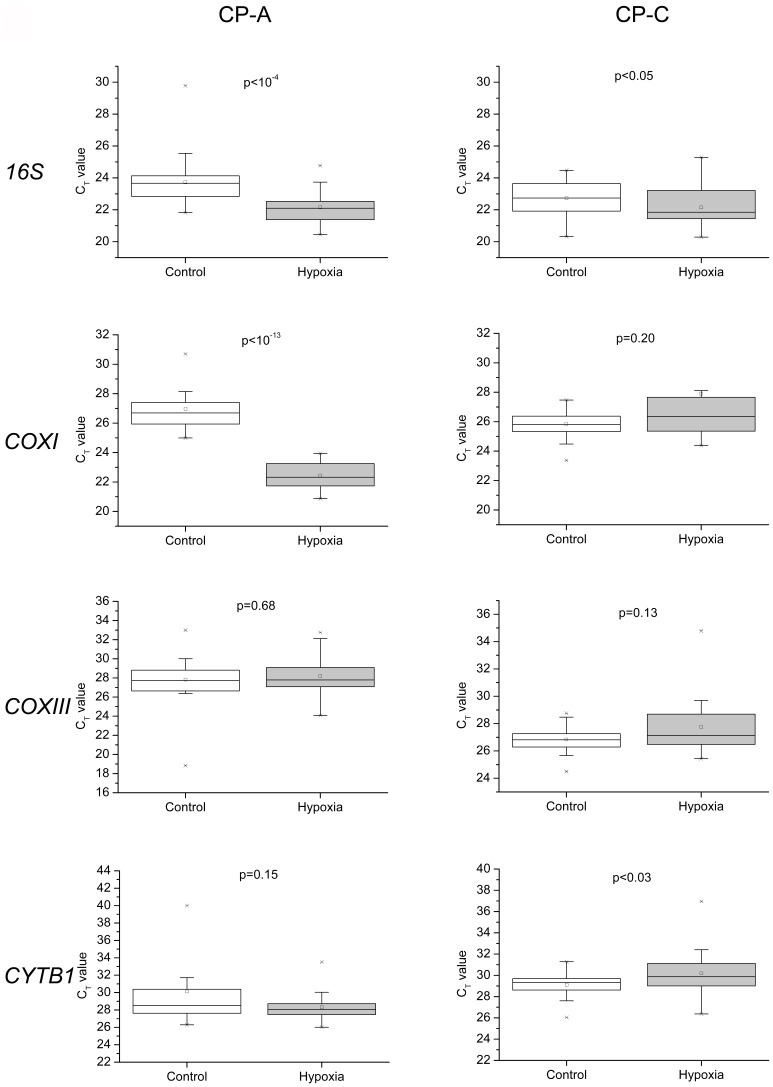
Expression levels of mitochondrially encoded genes (*16s rRNA* and *COXI*) in single control and hypoxia-treated CP-A and CP-C cells. Box plots of single-cell gene-expression levels and *p*-values associated with the differences between normoxic and hypoxic conditions in the two cell lines. The box chart shows following statistical values: Open square – mean, solid line – median, upper and lower box lines – the 75th and 25th percentiles, respectively, upper and lower whiskers – the 95th and 5th percentiles, respectively, x – maximal and minimal values.

**Figure 4:**
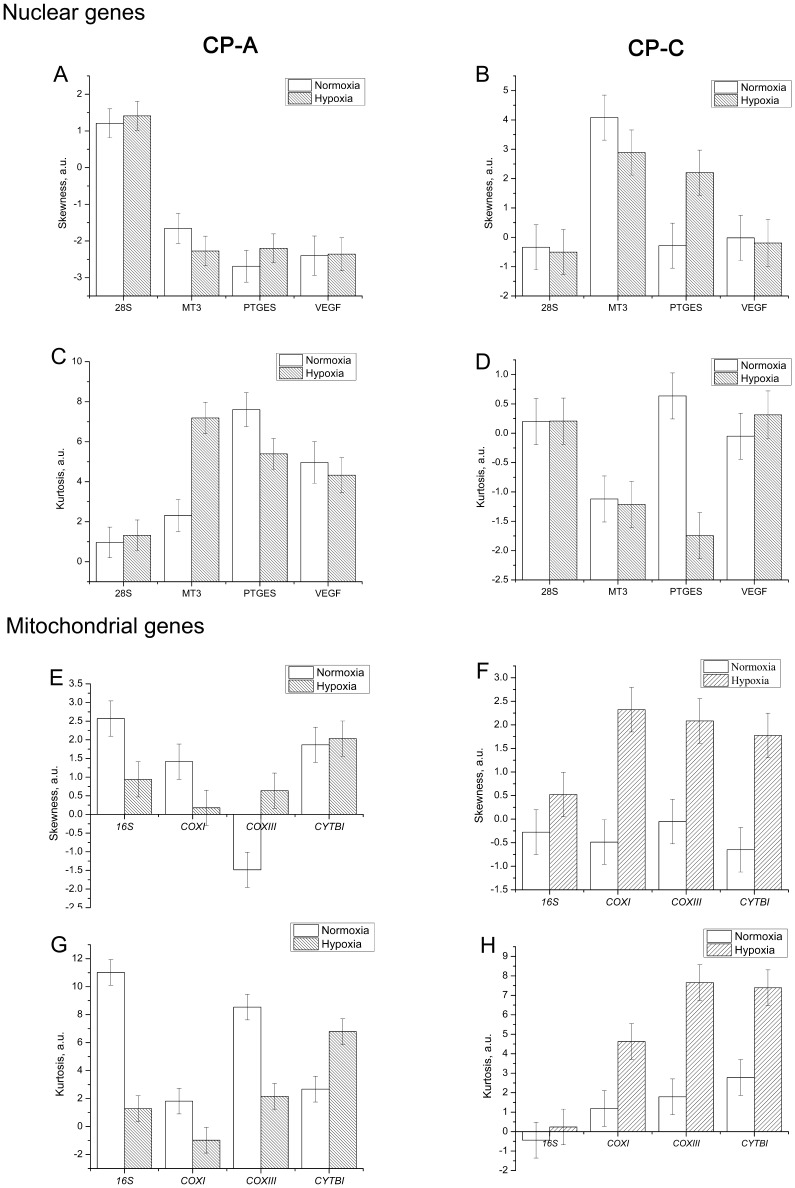
Single-cell expression levels distributions parameters of the studied mitochondrial and nuclear genes in normoxic and hypoxic metaplastic (CP-A) and dysplastic (CP-C) cells. Comparison of distribution skewness (A, B, E, F) and kurtosis (C, D, G, H) values of the single-cell gene transcription level distributions between normoxic and hypoxic conditions. A clear trend of increased both skewness and kurtosis of mitochondrially-encoded gene expression distributions can be seen in CP-C cells in response to hypoxia, but is absent in CP-A cells. Among the studied nuclear genes only the PTGES gene showed significant changes in skewness and kurtosis parameters in CP-C cells.

For gene expression analysis at the single-cell level, a total of 24 cells of each cell type and condition (normoxia and hypoxia) were isolated. The results showed a high degree of cell-to-cell variability in the expression levels of all four mitochondrial genes in cells of both types (Fig. 2–4). For instance, Ct values of 16s rRNA in CP-A cells ranged from 20 to 26 (reaching 30 in one case), and the range in CP-C cells was between 20 and 25. Ct values of *COXI*, *COXIII* and *CYTBI* in both cell lines were between 20 and 35 with average Ct values of 22.4∼27.9, 26.8∼28.2, and 28.3∼30.2, respectively (Fig. 2, 3). A significant reduction in Ct value of the 16s rRNA gene (*p*<0.05, n = 24) was observed in hypoxic CP-A single cells, while only slight but statistically significant change was detected in hypoxic CP-C cells (*p*<0.05, n = 24) (Fig. 3, *16s rRNA*). Similarly, a significant decrease in Ct value for *COXI* (*p*<0.001, n = 24) was observed in CP-A, but not in CP-C cells under hypoxic conditions as compared to normoxia (Fig. 2, 3, *COXI*). Since lower Ct values mean higher mRNA copy numbers, 16s rRNA and *COXI* mRNA levels in CP-A cells were significantly up-regulated by hypoxia, indicating that both genes in CP-A cells respond to hypoxia. Interestingly, when the average Ct*_COXI_* values were normalized against the expression level of 16s rRNA Ct*_16S_*, a standard step for calculation of the relative mRNA levels in bulk cell samples, the reduction of *COXI* Ct values in response to hypoxia was not significant, which is consistent with the bulk-level gene expression analysis results of this study (Fig. S4). In contrast, a significant Ct value increase was detected in *CYTBI* (*p* = 0.03, n = 24) in CP-C cells but not in CP-A cells (*p* = 0.43, n = 24) (Fig. 2, 3, *CYTB1*), which suggested a down-regulation by hypoxia of this mitochondrially encoded gene in CP-C cells. It is interesting to note that the expression levels of 16S rRNA in normoxic CP-C cells are higher than in normoxic CP-A cells (C_t(CPC)_ = 22.73 vs. C_t(CPA)_ = 23.66, p<0.006) and approaches the levels in hypoxic CP-A cells (C_t(CPA)_ = 22.09). However, our study has also shown that the average mtDNA copy numbers per cell are higher in CP-C cells (Fig. 1B, Table 1). Therefore, to make a direct comparison between the two cell lines possible, we calculated the relative differences in gene expression levels and normalized them against the average mtDNA copy numbers using the following equation:
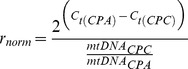
where mtDNA is the average mitochondrial DNA copy number per cell. By calculating r_norm_ we can determine the relative differences in gene expression levels between the two cell types per mtDNA molecule. This parameter can also be interpreted as a measure of “mtDNA activity” because it represents the ratio between the relative gene expression levels and mtDNA copy number. Utilizing this expression we find that under normoxic conditions 16S rRNA is expressed 33% higher per mtDNA molecule in CP-C cells than in CP-A cells. A similar result is valid for *COXI* (28% higher in CP-C than CP-A) and *COXIII* (32%), whereas *CYTBI* exhibits 2.45-fold lower expression per mtDNA molecule in CP-C compared with CP-A cells. Furthermore, we observe marked differences in the gene expression distribution parameters–skewness and kurtosis–between hypoxia responses in CP-A and CP-C cells (Fig. 4). Although no clear trend in distribution skewness or kurtosis is obvious in response to hypoxia in CP-A cells (Fig. 4A, C, E, G), CP-C cells exhibit a clear and statistically significant increase in both skewness and kurtosis of the distributions of *COXI*, *COXIII* and *CYTBI* gene expression (Fig. 4F, H). On the other hand, except for *PTGES*, the studied nuclear genes did not show significant differences in the distribution parameters in CP-C cells (Fig. 4B, D). It is important to note that the average values of *COXI* and *COXIII* expression levels in normoxic and hypoxic CP-C cells were not significantly different at the bulk level (Fig. S4). This finding demonstrates again the utility of single-cell analyses to gain access to information unavailable otherwise.

#### Differential expression of nuclear hypoxia response genes

**Figure 5:**
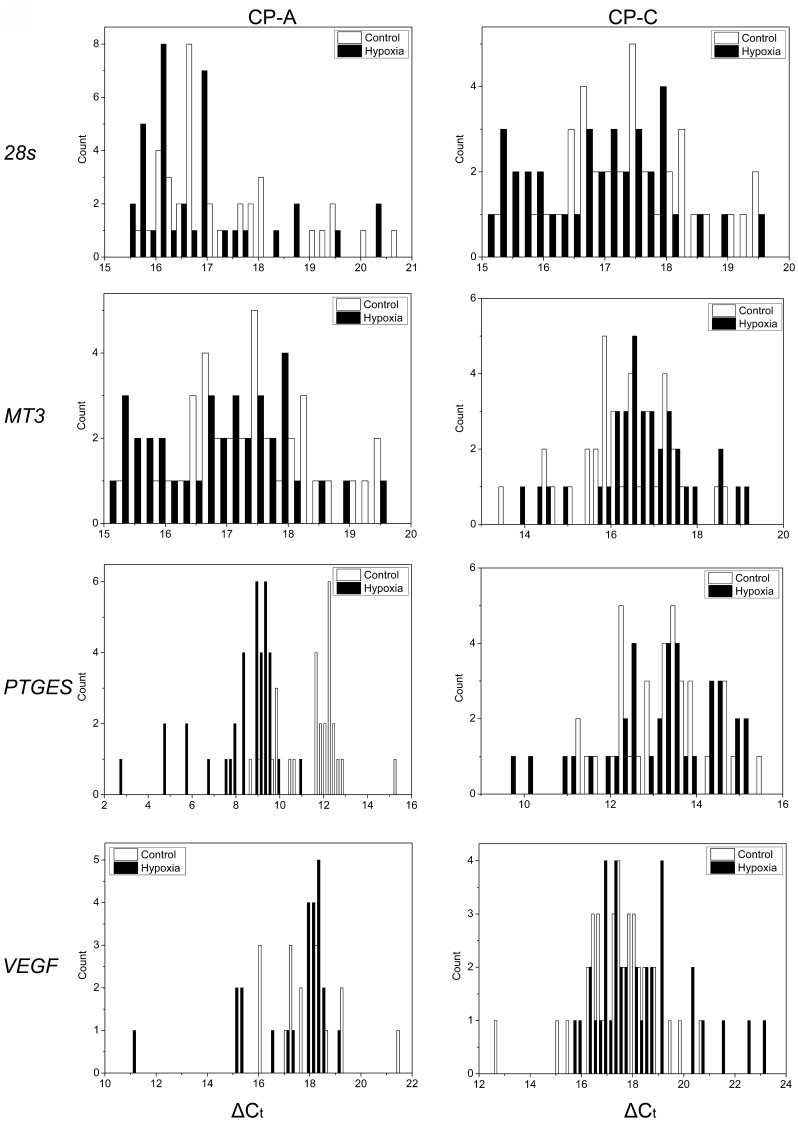
Expression levels of mitochondria and nucleus encoded genes in single control and hypoxia-treated CP-A and CP-C cells. Histograms of gene expression levels in control (*empty bars*) and hypoxia-treated (30 minutes, *solid bars*) cells. The distribution histograms were generated using the same bin size.

We have shown that gene expression levels determined from single-cell analysis were not always consistent with those obtained through bulk-cell analysis. Differential expression of two nuclear genes in response to hypoxia was observed between bulk CP-A and CP-C samples. In contrast to CP-A cells, where *MT3* was up-regulated ∼7-fold and *VEGF* showed no change, CP-C cells exhibited a ∼5 fold increase in *VEGF* expression while *MT3* showed no significant change (Fig. S4A, B). At the single-cell level we noticed that even *28s* rRNA, which is commonly used as internal reference in bulk cell studies, showed significant up-regulation in CP-A (*p*<0.05, n = 36), but not in CP-C (*p* = 0.05, n = 36) cells under hypoxia (Fig. 5, *28s*). Due to the varying levels of expression of *28s* rRNA, raw Ct values of hypoxia response genes instead of traditional delta Ct (normalized against a housekeeping gene) were used for further analysis.

**Figure 6:**
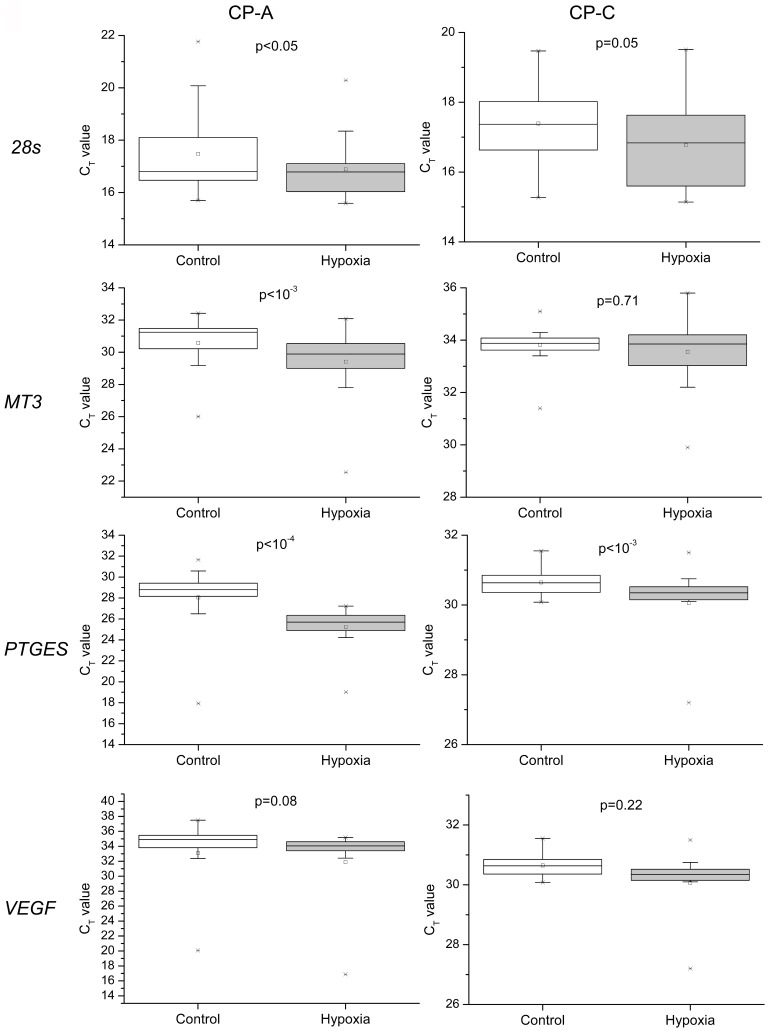
Expression levels of mitochondria and nucleus encoded genes in single control and hypoxia-treated CP-A and CP-C cells Box plots of single-cell gene expression levels and *p*-values (non-parametric Mann-Whitney test) associated with the differences between normoxic and hypoxic conditions. The box chart shows following statistical values: Open square – mean, solid line – median, upper and lower box lines – the 75th and 25th percentiles, respectively, upper and lower whiskers – the 95th and 5th percentiles, respectively, x – maximal and minimal values.

Although *28s* rRNA expression was detected in all single cells in the study, transcripts from the three other nuclear encoded genes were not always detected in CP-A cells from both control and hypoxic groups, most likely due to their low abundance. For instance, out of 36 single cells, *MT3* transcripts were detected in 33 control and 34 hypoxic single cells, *PTGES* in 29 and 36, and *VEGF* in 16 and 24. In CP-C single cells, however, except for *VEGF* (33 out of 36), transcripts from all genes were detected in all 36 single cells. Based on raw Ct values, two hypoxia response genes *MT3* (*p*<0.001, n = 33, 35 in control and hypoxic groups, respectively) and *PTGES* (*p*<10^−4^, n = 27, 36) had significantly increased expression levels in CP-A cells (Fig. 5, 6). Interestingly, in hypoxic CP-C cells only *PTGES* showed a significant up-regulation (*p*<0.001, n = 36, 36) while the other two, *VEGF* and *MT3*, did not (Fig. 5, 6). Differential *VEGF* gene expression under hypoxia was not observed in either CP-A or CP-C at single-cell levels. In contrast to the studied mitochondrial genes, we do not observe as many statistically significant differences in skewness and kurtosis parameters of the single cell gene expression distributions (Fig. 4A–D) between normoxic and hypoxic cells of both types. The distribution of the *PTGES* gene exhibited statistically significant increases in both skewness and kurtosis values in hypoxic vs. normoxic CP-C cells. The distribution shapes of the other three genes’ expression levels did not show statistically significant alterations in response to hypoxia. In CP-A cells only the *MT3* gene showed a markedly increased kurtosis in response to hypoxia. All other genes did not show significant changes in single-cell expression distribution parameters.

## Discussion

The finding of significantly elevated levels of mtDNA in CP-C compared to CP-A cells (Fig. 1, Table 1) is important as it bears potential functional relevance. Interestingly, both increased and decreased amounts of mtDNA have been reported in different types of human solid tumor cells. For example, reduced mtDNA levels have been found in prostate carcinoma cells and associated with invasive phenotype [50], a downregulation of mitochondrial biogenesis was correlated with invasive breast cancer [51] and ovarian cancer progression [52]. On the contrary, elevated amounts of mtDNA have been reported in prostate [53], pancreatic [54], head and neck cancer, gliomas [55], and found to positively correlate with clinicopathological stage in colorectal cancer [56], Shapovalov et al. have shown increased mtDNA levels and decreased respiration rates in aggressive osteosarcoma cells as compared to osteoblasts or benign osteosarcoma cells [57]. Furthermore, increased mtDNA levels have been associated with the risk of developing breast cancer [58] whereas increases in mtDNA copy numbers have been reported in progression from normal to pre-malignant to malignant progression in endometrium [59], carcinogenesis of head and neck cancers [60] and in the progression of esophageal squamous cell carcinoma [61]. While the functional role of increased mtDNA levels in pre-malignant to malignant progression remains to be elucidated, upregulation of the mitochondrial biogenesis was suggested to serve as a compensatory mechanism for impaired mitochondrial function due to mtDNA damage in pre-malignant lesions [60], [61]. It is thus possible that dysplastic esophageal epithelial cells upregulate mitochondrial biogenesis to increase the overall availability of template for transcription, which in turn should increase the net amount of mitochondrial mRNA and the corresponding protein levels to maintain mitochondrial function. We note that only single-cell level analysis revealed that mtDNA levels between CP-A and CP-C cells are significantly different, whereas bulk-level analysis showed no significant difference (Fig. S3). The observed higher asymmetry and peakedness values of mtDNA content distribution in CP-A cells are indicative of a higher degree of deviation from normal distribution CP-A compared to CP-C cells. While functional relevance of this finding remains to be elucidated, the result itself is interesting as it indicates population-level differences between the two stages in pre-malignant BE. It is possible that due to selective pressure conferred by the bile and acid reflux in the esophagus, one or several subclones in the more heterogeneous population of metaplastic (CP-A) cells are selected for resulting in a less heterogeneous, closer to normal distribution profile of mtDNA content in the more advanced, dysplastic stage (CP-C cells) of BE. Studies focusing on heteroplasmy of mitochondrial DNA at the single-cell level would need to be conducted to provide more detailed insight into this finding. Nevertheless, the finding of elevated mtDNA levels in dysplastic BE cells indicates that pre-malignant BE progression is similar to other types of progression (ESCC and head and neck cancer) and that it may be used as a biomarker for early detection of dysplasia in different tissue types.

The mitochondrial membrane potential (MMP) measurements revealed markedly higher relative values in normoxic CP-C (1.86±0.41) compared with CP-A (0.80±0.17) cells (Fig. 1, Table 2). When normalized against the mtDNA copy number, the average relative MMP values are about 37% higher in CP-C than in CP-A cells. This result shows that on average per mtDNA molecule the dysplastic cells maintain higher MMP values than the metaplastic cells. Higher MMP levels can result from a decrease in the ATP synthesis rate because the proton gradient is depleted at a lower rate. Our results on reduced oxygen consumption [33] and the increased MMP levels in CP-C cells support the notion of decreased ATPase and/or COX activities. Another potential mechanism contributing to the maintenance of the hyperpolarization of the inner mitochondrial membrane in CP-C cells could be the HIF-1a mediated replacement of the COX4-1 subunit with the COX4-2 isoform which is known to increase the efficiency of COX under hypoxic conditions [62], [63].

The analysis of image cytometry data has also revealed marked differences in the mitochondrial membrane potential (MMP) between the two cell types in response to hypoxia. The MMP in hypoxic CP-C cells was retained at markedly higher levels than in hypoxic CP-A cells. When normalized against the average mtDNA copy number per cell, the MMP values were about 28% higher per mtDNA molecule in CP-C vs. CP-A cells. The regulatory role of mitochondrial function and MMP in hypoxia response is of critical importance [64]. The maintenance of high MMP levels in cancer cells has been recognized as one of the hallmarks of cancer and is thought to be necessary to enable several vital processes to take place in response to low oxygen availability in tissues. A direct correlation between increased MMP levels and malignant phenotype has been reported in the literature [65]–[67] and attributed to an increased capacity of tumor cells to respond to hypoxia by avoiding apoptosis [39], [66], [68], evade anoikis, prevent ATP consumption by the hydrolytic activity of the ATPase, and excessive ROS production [69], and invade the basement membrane [66]. It is possible that a combination of these three mechanisms and maybe others contribute to the observed high MMP levels in CP-C cells. We observe markedly higher kurtosis values of the MMP distribution in CP-C cells which suggests higher MMP heterogeneity of mitochondria in dysplastic (CP-C) than metaplastic (CP-A) BE cells under normoxic conditions. This finding is in contrast to lower mtDNA copy number heterogeneity observed in CP-C as compared with CP-A cells. It suggests that opposite to heterogeneity in mtDNA copy numbers, mitochondrial function variability is more pronounced in dysplastic than in non-dysplastic cells. Furthermore, this finding implies that at the population level variability in mtDNA amount per cell does not necessarily correlate with the variability in the mitochondrial function in terms of MMP. It is thus likely that mtDNA heteroplasmy plays a significant role in modulating mitochondrial activity at the single-cell level. In addition to the observed differences under normoxia, we noticed a significant difference in MMP distribution parameters in response to hypoxia. While kurtosis increased from 0.07 to 1.11 in CP-A cells as a result of hypoxia, a significant decrease (from 0.94 to 0.09) was observed in CP-C cells. It is possible that even though mitochondrial activity levels in CP-C cells are more heterogeneous under normal conditions, the hypoxic insult results in a close to normal distribution of MMP, potentially indicating a fairly uniform response to low oxygen among individual dysplastic cells. This would suggest lower population-level heterogeneity in terms of hypoxia response in dysplastic compared to non-dysplastic cells and would support the working hypothesis of clonal selection as a result of acid/bile reflux in BE. At this point it is somewhat difficult to provide a more detailed insight into functional relevance of the hypoxia response differences, and studies focusing on genomic and/or mtDNA sequencing and functional phenotyping of individual cells from particular subpopulations are needed. However, these differences could be used as potential biomarkers for early detection of pre-malignant stages in BE and may offer new therapeutic targets for early prevention of EAC.

CP-A cells respond to hypoxia with a marked upregulation of *COXI* and *16S* rRNA, whereas CP-C cells showed only slight, although statistically significant, differences in the expression levels of the *16S* and *CYTBI* genes before vs. after hypoxia treatment (Fig. 2, 3). *COXI* is actively involved in the electron transfer step from reduced cytochrome c to the binuclear heme-copper center [70]. Hence, the upregulation of *COXI* in hypoxic CP-A cells may represent an attempt by the cell to adjust to the diminishing availability of oxygen. It is likely that the upregulation of *COXI* is necessary to increase the overall oxidation rate of cytochrome c and in this way compensate for the reduced oxygen levels and maintain ATP production levels. The absence of a similar response in dysplastic (CP-C) cells, which are further progressed toward malignancy, implies that the dysplastic BE cells are most likely less dependent on OXPHOS. Furthermore, the hypoxia response mechanisms that are in place in metaplastic (CP-A) cells are absent or inactive to produce a significant upregulation of *COXI* expression levels in dysplastic (CP-C) cells. Irrespective of the mechanism underlying the differential expression of *COXI* and *COXIII*, it is obvious that in comparison to CP-C cells, CP-A cells are more sensitive to short-term hypoxia and upregulate at least two (*16S* and *COXI*) of the four studied mitochondrially encoded genes. The absence of a substantial upregulation of *16s* rRNA transcription in CP-C cells provides another important insight: Mitochondrial transcription machinery in CP-C cells is either not being activated or activated to a much lower extent than in CP-A cells in response to short-term hypoxia. This finding is further corroborated by the absence of significant increases in the other three studied mitochondrial genes in CP-C cells. The transcription level analysis of the four nuclear-encoded genes (*28S*, *MT3*, *PTGES*, and *VEGF*) provides more support for this notion (Fig. 5, 6). The absence of a significant response in transcription of the four studied nuclear genes in hypoxic CP-C cells indicates that CP-C cells are less sensitive to short-term oxygen deprivation than CP-A cells. To identify and characterize possible changes in gene expression level heterogeneity profiles in response to hypoxia, we conducted a statistical analysis of the single cell data. In case of mitochondrial gene expression we observe a distinct trend in increased skewness and kurtosis values in three of four studied mitochondrial genes of hypoxic CP-C cells. This trend is either reversed or absent in CP-A cells (Fig. 4). In our case this means that the response to hypoxia in CP-C cells manifests in a more asymmetric (non-normal) distribution of the expression levels of the three genes. The shape of the mRNA distribution, and its parameters such as skewness and kurtosis, depend strongly on the transcription regulation mechanism. Studies on the dynamics of the single-cell mRNA expression levels have been conducted in the past [71]–[73]. Two different types of mRNA temporal expression behavior have been identified. One is a result of the constantly active, or non-bursting, transcription machinery with intrinsic noise and stochastic behavior and exhibits a normal, or close to normal, distribution of mRNA copy numbers over time [72]. The second distinct type of transcriptional behavior dynamics is caused by repeated activation/inactivation cycles of the transcription by several different mechanisms, including transcription factor binding/unbinding, chromatin remodeling events and mRNA elongation regulation [73]. When, for example, a transcription factor binds to its cognitive site on the DNA molecule, the transcription of the corresponding gene is activated and remains active until the transcription factor dissociates. The switching between the active and inactive transcriptional states results in a burst-like transcription pattern with a strongly asymmetric temporal distribution of mRNA copy numbers in a cell. Even though the average mRNA levels can be similar between these two cases, the shape parameters of the two distributions differ markedly. Whereas the non-bursting dynamics can produce symmetric distributions of mRNA levels in time with low skewness and kurtosis (mesokurtic) values, the bursting mRNA expression dynamics results in highly asymmetric distributions with significantly higher skewness and kurtosis levels. In this study we measured “snapshots” in time of mRNA expression levels in individual cells. In this regard, the mRNA distributions represent spatial rather than temporal profiles since the cells to be analyzed were picked at random from different locations within the cell population. However, because the cell population itself was not synchronized with regard to gene transcription, the mRNA levels in randomly picked cells reflect the temporal behavior of the transcription activity. Due to close qualitative similarities of the mRNA distribution skewness and kurtosis observed in this study with the temporal profiles of mRNA expression reported earlier, we posit that molecular mechanisms based on non-bursting and bursting mRNA expression regulation are at play in hypoxia response. It is likely that while normoxic CP-A cells exhibit more burst-like expression of mitochondrial encoded genes *16S*, *COXI* and *COXIII*, the transcription dynamics changes to more symmetric, constitutive (non burst-like) behavior. On the contrary, except for the *16S* gene, CP-C cells show a qualitatively opposite behavior – symmetric, non-bursting-like distribution under normoxia and burst-like characteristics under hypoxic conditions. At this point it is difficult to interpret the functional and mechanistic implications of the two types of behavior on cellular phenotype, and more detailed studies need to be conducted to specifically address this intriguing point. We note, however, that although marked changes in mRNA level distribution were observed in hypoxic CP-C cells, the average mRNA levels did not change significantly. This parallels the findings of several earlier studies which demonstrated the possibility of different mRNA distribution characteristics with identical average values. The finding of differing distribution parameters demonstrates the utility of single-cell studies to reveal dynamic events which would otherwise be averaged out in bulk-cell approaches.

Combining the mitochondrial gene expression levels with the higher relative MMP levels observed in hypoxic CP-C as compared to hypoxic CP-A cells, it appears likely that dysplastic esophageal epithelial cells are primed for short-term hypoxia and are much better adapted to brief periods of severe oxygen deprivation than metaplastic epithelial cells. The findings of this and other studies indicate that in dysplastic esophageal epithelial cells the most likely mechanisms contributing to the maintenance of high MMP levels are similar to those reported in cancer cells of different types. This implies that in this early pre-malignant stage that predisposes to carcinogenesis, cells already exhibit several important characteristics commonly found in cancer cells.

## Conclusion

In summary, using single cell mtDNA copy number and gene expression techniques, we find that the dysplastic CP-C cells exhibit several important characteristics commonly found in many types of cancer cells, namely 1) elevated amounts of mtDNA, 2) high MMP levels under normoxia, and 3) the ability to maintain relatively high MMP values under hypoxia. Furthermore, compared to metaplastic cells, dysplastic cells are much less sensitive to oxygen deprivation in terms of transcriptional activity of the studied mitochondrial and nuclear genes. Taken together these findings indicate a much higher degree of resistance to short-term hypoxia, which is likely to take place in vivo, of dysplastic compared to metaplastic esophageal epithelial cells. The presence of the features characteristic of malignant cells indicates an early onset of phenotypic and biomolecular transformations typically found in cancer cells. Alterations in mitochondrial function appear to be critical in the pre-malignant transformation of esophageal epithelium. A deeper understanding of these early alterations may be crucial for finding new therapeutic targets. Because hypoxia resistance is associated with many types of tumors, studies like this may provide insights into molecular mechanisms of other tumor types, such as cervix, lung and breast, that have been shown to contain hypoxic regions.

## Supporting Information

Figure S1 Amplification plots and melting curves of mtDNA (HV1) and gene transcripts (*16s* rRNA, *COXI*, *COXIII*, *CYTBI*) using validated primers. 2 µL (1/20^th^) of DNaST solution of the total cDNA obtained from a single CP-A cell was used for each qPCR reaction shown. This includes three technical replicates and the no-template controls (NTC). Each panel shows real-time amplification signal curves obtained from a single cell and respective melting curves of the selected primers. A) Amplification plots of each primer pair; the insets are gel verification of qPCR products, insets indicated the 1.5% agarose gel electrophoresis results of qPCR products; B) Melting curves of each primer pair.
(TIF)

Figure S2 Standard dilution curves for single cell mtDNA copy number analysis.
(TIF)

Figure S3 qPCR results of average mtDNA copy number in CP-A and CP-C single cells at the bulk cell levels based on biological triplicates. p>0.05.
(TIF)

Figure S4 Response patterns of three mitochondrial genes and three nuclear hypoxia response genes in bulk CP-A and CP-C cells samples.
(TIF)

Methods S1 (DOCX)
