# Supplementary figures and images for: Single-Cell Analysis Reveals Early Manifestation of Cancerous Phenotype in Pre-Malignant Esophageal Cells
Source: PLoS One. 2013 Oct 8;8(10):e75365. doi: 10.1371/journal.pone.0075365 (PMC3792915; doi:10.1371/journal.pone.0075365)

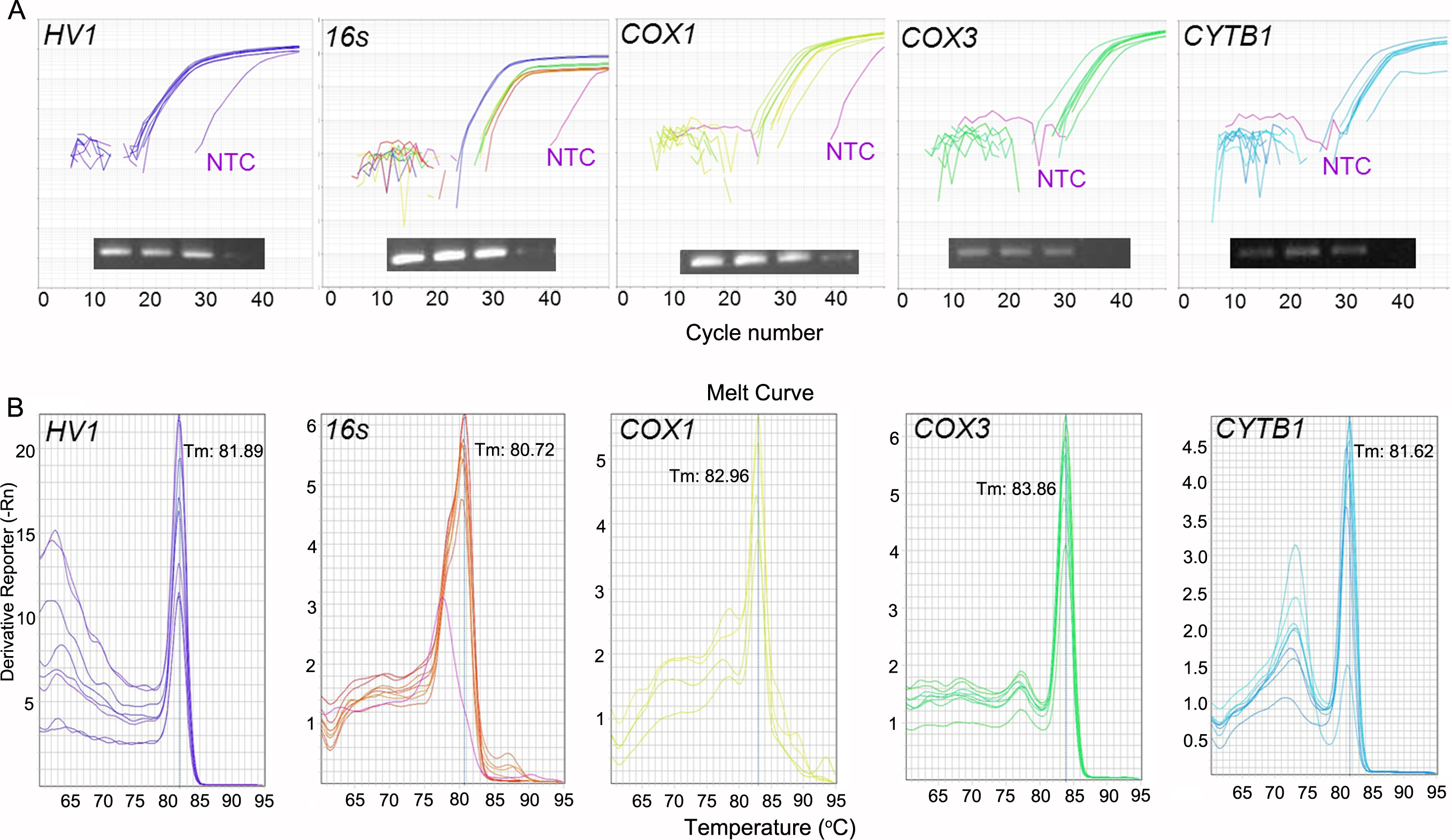

Supplement: Figure S1 — Amplification plots and melting curves of mtDNA (HV1) and gene transcripts (16s rRNA, COXI, COXIII, CYTBI) using validated primers. 2 µL (1/20th) of DNaST solution of the total cDNA obtained from a single CP-A cell was used for each qPCR reaction shown. This includes three technical replicates and the no-template controls (NTC). Each panel shows real-time amplification signal curves obtained from a single cell and respective melting curves of the selected primers. A) Amplification plots of each primer pair; the insets are gel verification of qPCR products, insets indicated the 1.5% agarose gel electrophoresis results of qPCR products; B) Melting curves of each primer pair. (TIF) [file pone.0075365.s001.tif]

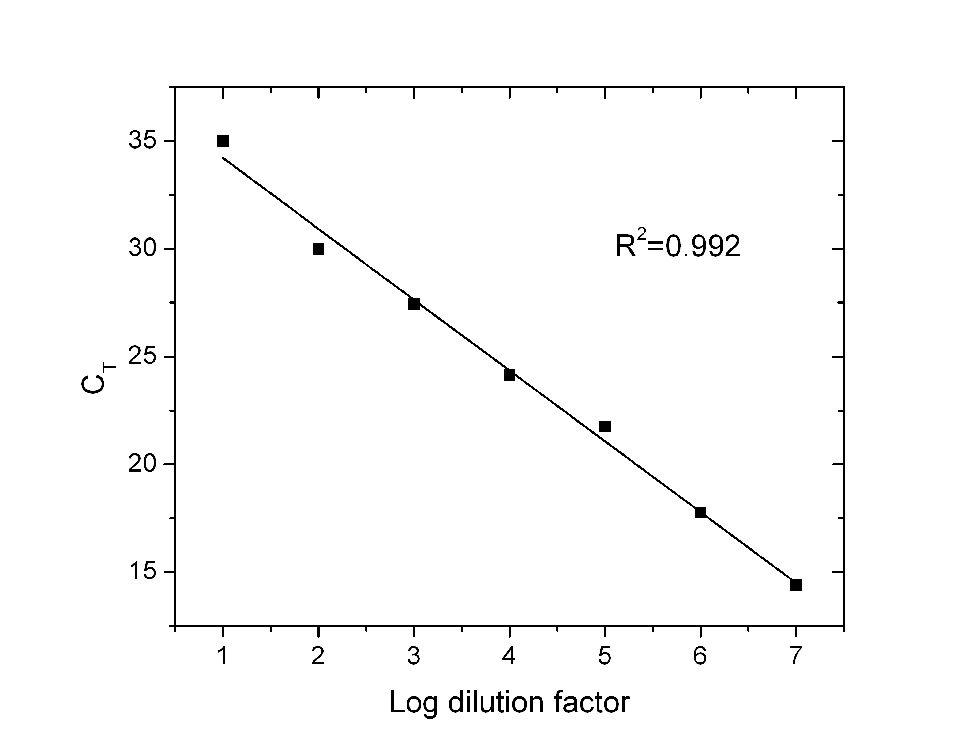

Supplement: Figure S2 — Standard dilution curves for single cell mtDNA copy number analysis. (TIF) [file pone.0075365.s002.tif]

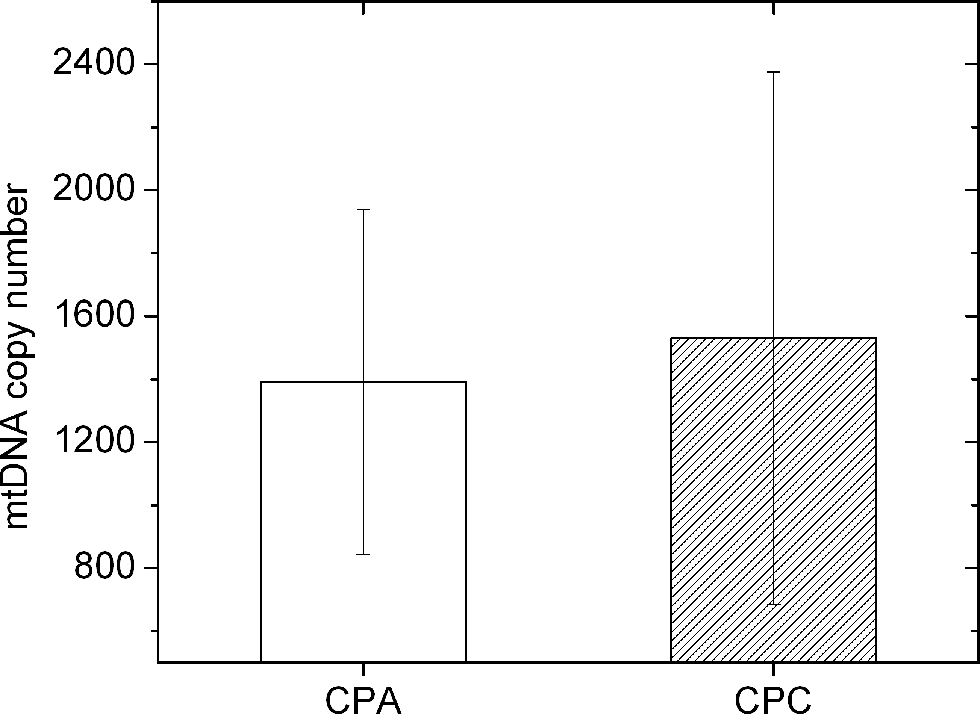

Supplement: Figure S3 — qPCR results of average mtDNA copy number in CP-A and CP-C single cells at the bulk cell levels based on biological triplicates. p>0.05. (TIF) [file pone.0075365.s003.tif]

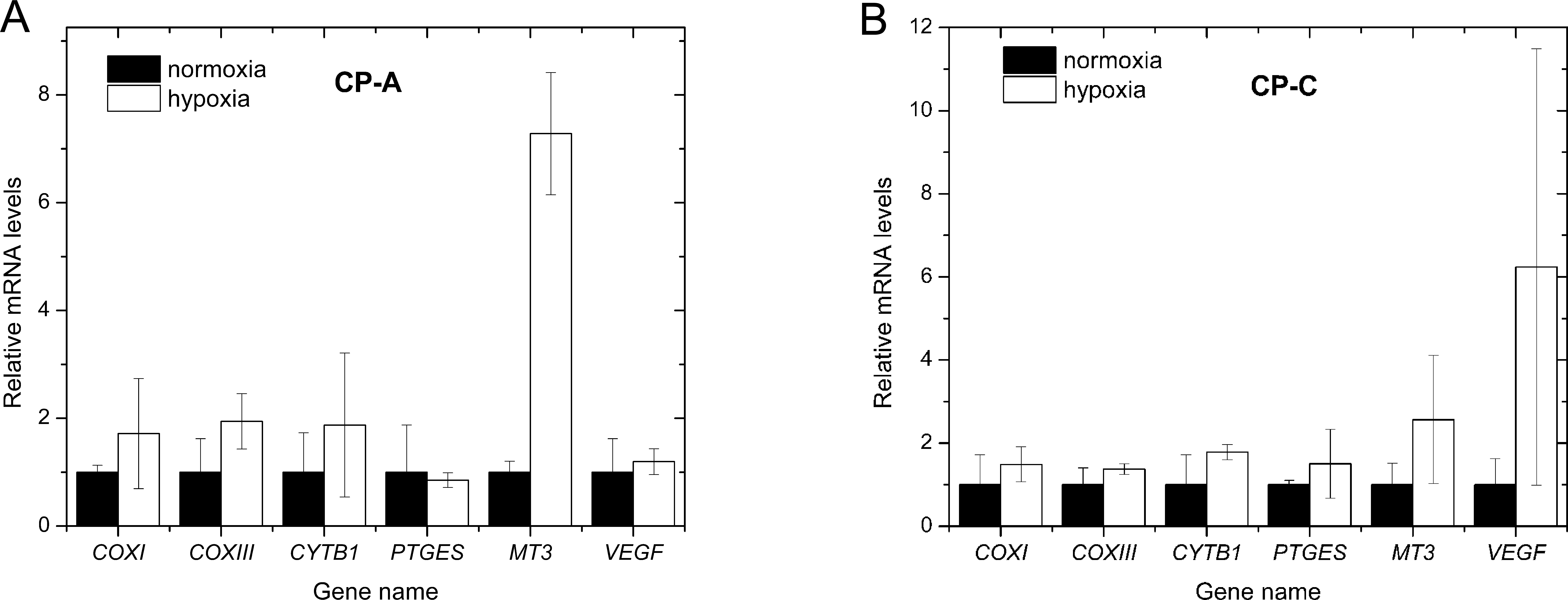

Supplement: Figure S4 — Response patterns of three mitochondrial genes and three nuclear hypoxia response genes in bulk CP-A and CP-C cells samples. (TIF) [file pone.0075365.s004.tif]
