# Supplementary material for: Single-Cell Analysis Reveals Early Manifestation of Cancerous Phenotype in Pre-Malignant Esophageal Cells
Source: PLoS One. 2013 Oct 8;8(10):e75365. doi: 10.1371/journal.pone.0075365 (PMC3792915; doi:10.1371/journal.pone.0075365)
Supplement: Methods S1 — (DOCX) [file pone.0075365.s005.docx]

**Supplementary materials**

**Methods**

**Single cell harvesting**

Suspended, flow-cytometry sorted G1 phase cells were used for single cell harvesting. Harvested single cells were loaded directly into a PCR cap containing 20 µL of 1X PBS buffer for gene expression analysis or 6 µL of DNaST buffer (NaCl: 0.135 M; Tris-HCl pH 8.0: 9 mM; dithiothreitol: 4.5 mM) (19) for mtDNA copy number measurements. To ensure the cell is fully covered with the buffer and is located at the bottom of the PCR tubes, the PCR tubes were briefly centrifuged in a “Quick-Spin” Minifuge (ISC BioExpress, Kaysville, UT, USA).

**Primers and qPCR**

Gene sequences were retrieved from GenBank. qPCR primers were designed using the PrimerExpress V2.0 software package (Applied Biosystems). Optimized primer oligos for single-cell analysis of the target genes were obtained from Invitrogen (Life Technologies, Grand Island, NY). HVI (forward: 5’-GCAGATTTGGGTACCACCCA, reverse: 5’-CGAGAAGGGATTTGACTGTAATGTG); Four mitochondrially encoded genes (16s rRNA forward: 5’-AGAAATTGAAACCTGGCGCA, reverse: 5’- TCGGTAGGTTTGTCGCCTCT; *COXI* forward: 5’-TCCTTATTCGAGCCGAGCTG, reverse: 5’- CACTATAGCAGATGCGAGCAGG; *COXIII* forward: 5’- AGCCCCTACCCCCCAATTAG, reverse: 5’- CGGAAATGGTGAAGGGAGACT, and *CYTBI* forward: 5’- GCCTGCCTGATCCTCCAAAT, reverse: 5’- GCAAGCAGGAGGATAATGCC) and four nuclear genes (28s rRNA, VEGF, MT3, and PTGES)(primers sequences as (Ref. 20)) were chosen. For mtDNA copy number and mitochondrial- and nuclear-encoded gene expression level analysis, both a positive control (serially diluted DNA or cDNA derived from bulk cells) and a negative control (distilled H_2_O instead of cell lysate or cDNA as template) were included in each qPCR run for quality control. Representative amplification plots for the selected mitochondrial genes are shown in Suppl. Fig. 1. Samples with detection threshold (Ct) values indistinguishable from the no-template controls (NTC) were considered failed and were removed from further data analysis. Samples with standard deviations of the technical replicates smaller than 5% were considered successful. In most qPCR reactions NTC generated no amplification products (see *COXIII* and *CYTBI* samples in Suppl. Fig. 1). In several reactions NTC amplification product was detected but with significantly higher Ct values (at least 5 or 6 cycles) than those of the single-cell samples (see 16s rRNA and *COXI* in Suppl. Fig. 1). The bands containing products of the right size (insets in Supple. Fig. 1) were gel-purified and sequenced. The product sequences were confirmed using BLAST annotation against the NCBI GenBank database.

**Mitochondrial membrane potential determination**

Mitochondrial membrane potential (MMP) was quantified with confocal microscopy analysis using the potentiometric dye JC-1 (100 ng/mL) as fluorophore and published staining protocols

Briefly, 80% confluent CP-A and CP-C cells in 24-well plates were incubated in fresh serum-free medium w/o serum containing 100 ng/mL JC-1 for 15 min. The cells were washed twice with fresh medium w/ serum, and fluorescence micrographs of cells were acquired on a confocal microscope (Cs1, Nikon, Melville, NY). JC-1 fluorescence intensity signals of about 100 randomly selected cells per cell type and treatment were analyzed using Nikon Elements analysis software (v. 3.2, Nikon).

**Figure legends**

**Supplementary Fig. 1.** Amplification plots and melting curves of mtDNA (HV1) and gene transcripts (*16s* rRNA, *COXI*, *COXIII*, *CYTBI*) using validated primers. 2 µL (1/20^th^) of DNaST solution of the total cDNA obtained from a single CP-A cell was used for each qPCR reaction shown. This includes three technical replicates and the no-template controls (NTC). Each panel shows real-time amplification signal curves obtained from a single cell and respective melting curves of the selected primers. A) Amplification plots of each primer pair; the insets are gel verification of qPCR products, insets indicated the 1.5% agarose gel electrophoresis results of qPCR products; B) Melting curves of each primer pair.

**Supplementary Fig. 2.** Standard dilution curves for single cell mtDNA copy number analysis.

**Supplementary Fig. 3.** qPCR results of average mtDNA copy number in CP-A and CP-C single cells at the bulk cell levels based on biological triplicates. p > 0.05.

**Supplementary Fig. 4.** Response patterns of three mitochondrial genes and three nuclear hypoxia response genes in bulk CP-A and CP-C cells samples.
